# Evaluation of cannabidiol’s inhibitory effect on alpha-glucosidase and its stability in simulated gastric and intestinal fluids

**DOI:** 10.1186/s42238-021-00077-x

**Published:** 2021-06-23

**Authors:** Hang Ma, Huifang Li, Chang Liu, Navindra P. Seeram

**Affiliations:** Bioactive Botanical Research Laboratory, Department of Biomedical and Pharmaceutical Sciences, College of Pharmacy, University of Rhode Island, 7 Greenhouse Rd, Kingston, RI 02881 USA

**Keywords:** Cannabidiol, *Α*-Glucosidase, Type II diabetes mellitus, Gastric fluid, Intestinal fluid

## Abstract

**Objective:**

Cannabidiol (CBD) has been reported to have anti-diabetic effects in pre-clinical and clinical studies but its inhibitory effects on *α*-glucosidase, a carbohydrate hydrolyzing enzyme, remain unknown. Herein, we evaluated CBD’s inhibitory effects on *α*-glucosidase using in vitro assays and computational studies.

**Methods:**

CBD’s inhibitory effect on *α*-glucosidase activity was evaluated in a yeast enzymatic assay and by molecular docking. The stability of CBD in simulated gastric and intestinal fluids was evaluated by high-performance liquid chromatography analyses.

**Results:**

CBD, at 10, 19, 38, 76, 152, 304, 608, and 1216 μM, inhibited *α*-glucosidase activity with inhibition of 17.1, 20.4, 48.1, 56.6, 59.1, 63.7, 74.1, and 95.4%, respectively. Acarbose, the positive control, showed a comparable inhibitory activity (with 85.1% inhibition at 608 μM). CBD’s inhibitory effect on *α*-glucosidase was supported by molecular docking showing binding energy (-6.39 kcal/mol) and interactions between CBD and the *α*-glucosidase protein. CBD was stable in simulated gastric and intestinal fluids for two hours (maintained ≥ 90.0%).

**Conclusions:**

CBD showed moderate inhibitory effect against yeast *α*-glucosidase activity and was stable in gastric and intestinal fluids. However, further studies on CBD’s anti-*α*-glucosidase effects using cellular and in vivo models are warranted to support its potential application for the management of type II diabetes mellitus.

## Introduction

Increasing legalization and social perceptions of medical use of cannabis raises the issue of patients using cannabis and its derivatives, such as cannabidiol (CBD), to self-medicate for certain medical conditions including diabetes. Claims that cannabis derivatives have beneficial effects on diabetic conditions are easily accessed by the public. However, these claims of cannabis combating diabetes are not substantially supported by medical research published in peer-reviewed journals. To date, only one randomized, double-blind, placebo-controlled, parallel group pilot study reported the investigation of CBD’s efficacy on glycemic parameters in patients with type II diabetes (Jadoon et al. 2016). In this study, although CBD ameliorated diabetic conditions including decreased resistin and increased glucose-dependent insulinotropic peptide, it showed no effect on lowering glycemic parameters in patients. Another challenge to understanding the effects of cannabis and its derivatives on diabetic conditions is inadequate basic experimental research including biochemical and biological studies. There are only few published studies reporting the effects of CBD as an intervention for diabetes in rodent models. Two studies showed that CBD was able to arrest the onset and progress of diabetes in non-obese diabetes-prone mice (Weiss et al. 2008, 2006). In addition, limited studies reported CBD’s ameliorative effects against diabetic complications including endothelial dysfunction (Stanley et al. 2013) and early pancreatic inflammation (Lehmann et al. 2016) in rodent diabetes models, as well as diabetic cardiomyopathy in primary human cardiomyocytes (Rajesh et al. 2010). However, to date, CBD’s anti-diabetic mechanisms, such as the identification of its biochemical targets including enzymes, are not reported. Enzymes mediating the metabolism of carbohydrates are particularly critical for diabetes management. For instance, *α*-glucosidase is one of the glycoside hydrolase enzymes that break down complex carbohydrates (e.g. starch and sucrose) into sugar monomers (i.e. glucose), which can then be absorbed through the intestine (Bischoff 1995). Therefore, *α*-glucosidase inhibitors are regarded as a therapeutic approach for type II diabetes mellitus management because of its capacity for decreasing postprandial blood glucose levels. The *α*-glucosidase inhibitor, acarbose (trade name, Glucobay or Precose), is used as a clinical treatment for glycemic control over hyperglycemia. Our group has had a long research interest in the identification of *α*-glucosidase inhibitors from medicinal plants (Liu et al. 2018; Omar et al. 2012; Wan et al. 2012; Yuan et al. 2013, 2012b, 2012a; Zhang et al. 2015) and the evaluation of these natural products for their potential mechanisms of inhibition (Ma et al. 2015). To date, the anti-*α*-glucosidase effects of cannabis and its derivatives remain unknown. Herein, we aimed to evaluate the inhibitory effects of CBD on *α*-glucosidase activity using an enzyme (from recombinant yeast) based in vitro assay. In addition, CBD’s inhibitory effect on *α*-glucosidase was studied by computational docking experiment and its stability was evaluated in simulated gastric and intestinal fluids.

## Materials and methods

### Chemicals

Cannabidiol (CBD), acarbose, dimethyl sulfoxide (DMSO), *α*-glucosidase (EC 3.2.1.20) from *Saccharomyces cerevisiae*, *p*-nitrophenyl-α-D-glucopyranoside (*p*NPG), methanol (analytical grade), were obtained from Sigma Aldrich (St. Louis, MO, USA). Simulated gastric fluid (CAT# 7108–16) and intestinal fluid (CAT# 7109.75–16) were obtained from RICCA Chemical Company (Arlington, TX, USA).

### α-Glucosidase inhibition assay

*α*-Glucosidase inhibition assay was carried out using previously reported method (Ma et al. 2015). In brief, CBD stock solution was prepared in DMSO (10 mg/mL) and diluted to desired concentrations with phosphate buffer (0.1 M, pH 6.8). A mixture of test sample (10 μL), phosphate buffer (80 μL), and yeast *α*-glucosidase (0.5 U/mL; 10 μL) was incubated in a 96-well plate at room temperature for 10 min followed by adding *p*NPG (1 M; 100 μL) to each well. The reaction mixtures were incubated at room temperature for 30 min before the absorbance was recorded at wavelength of 405 nm with a micro-plate reader (SpectraMax M2, Molecular Devices Corp., operated by SoftmaxPro v.4.6 software, Sunnyvale, CA, USA). *α*-Glucosidase inhibitory activity was expressed as inhibition% as compared to the control group. Each sample was tested three times, each in three replicates (n = 3), and data were shown as mean ± standard deviation (S.D). Statistical analysis was performed using GraphPad Prism 7 (GraphPad Software; La Jolla, CA, USA) using one-way analysis. A *p-*value that less than 0.001 (***) or 0.0001 (****) was considered as a statistical significance between the control group and the experimental groups.

### Molecular docking

The 3D structure of *α*-glucosidase from *Saccharomyces cerevisiae* were retrieved in PDB format from the RCSB protein data bank (www.rcsb.org; PDB ID: 3A4A) (Ma et al. 2015). Chimera 1.11.2 was applied to delete the ions, solvent, and ligands from target protein and to save the protein portion of 3A4A in pdb format. The 3D structural coordinates of CBD were obtained from the human metabolome database (www.HMDB.ca). AutodockTools 1.5.6 was used to perform molecular docking using Autodock 4.2 algorithm (Molecular Graphics Laboratory, The Scripps Research Institute, La Jolla, USA). Files of CBD and 3A4A were converted into pdbqt format and then a grid parameter file was prepared as a glg file in AutoGrid 4 software. Docking parameter file was prepared for the final docking and Autodock 1.5.6 software was used to perform molecular docking.

### High-performance liquid chromatography (HPLC) analysis of CBD in simulated gastric and intestinal fluids

CBD was prepared in simulated gastric and intestinal fluids according to a previously reported method (Merrick et al. 2016). Briefly, a CBD stock solution (100 mg/mL) was prepared in DMSO and aliquoted into phosphate buffer (pH = 6.8; as the control group) or into simulated gastric and intestinal fluids (as the experimental groups) to reach a final concentration of 200 μg/mL (equivalent to 636 μM). The CBD solutions were incubated at 37 °C in glass vials covered with aluminum foil to avoid light for two hours and then sampled for HPLC analysis. Analytical HPLC was performed on a Hitachi Elite LaChrom system consisting of a L2130 pump, L-2200 autosampler, and a L-2455 diode array detector (220–400 nm), operated by EZChrom Elite software (Hitachi High Technologies America; Dallas, TX, USA). All analyses were performed with an Alltima (Grace Davidson Discovery Science, Deerfield, IL, USA) C_18_ column (250 × 4.6 mm i.d., 5 μm) and the HPLC solvent system consisted of 0.1% trifluoroacetic acid in water (B)/methanol (A) linear gradient as follows: 1–19 min,80% A; 19–20 min, 80–100% A; 20–30 min, 100% A; 30.1–36 min, 80% with a total run time of 36 min, a flow rate of 0.75 mL/min, and an injection volume of 10 μL. The CBD content in the solutions were presented as the area under curve (AUC) value.

## Results and discussion

**Fig. 1:**
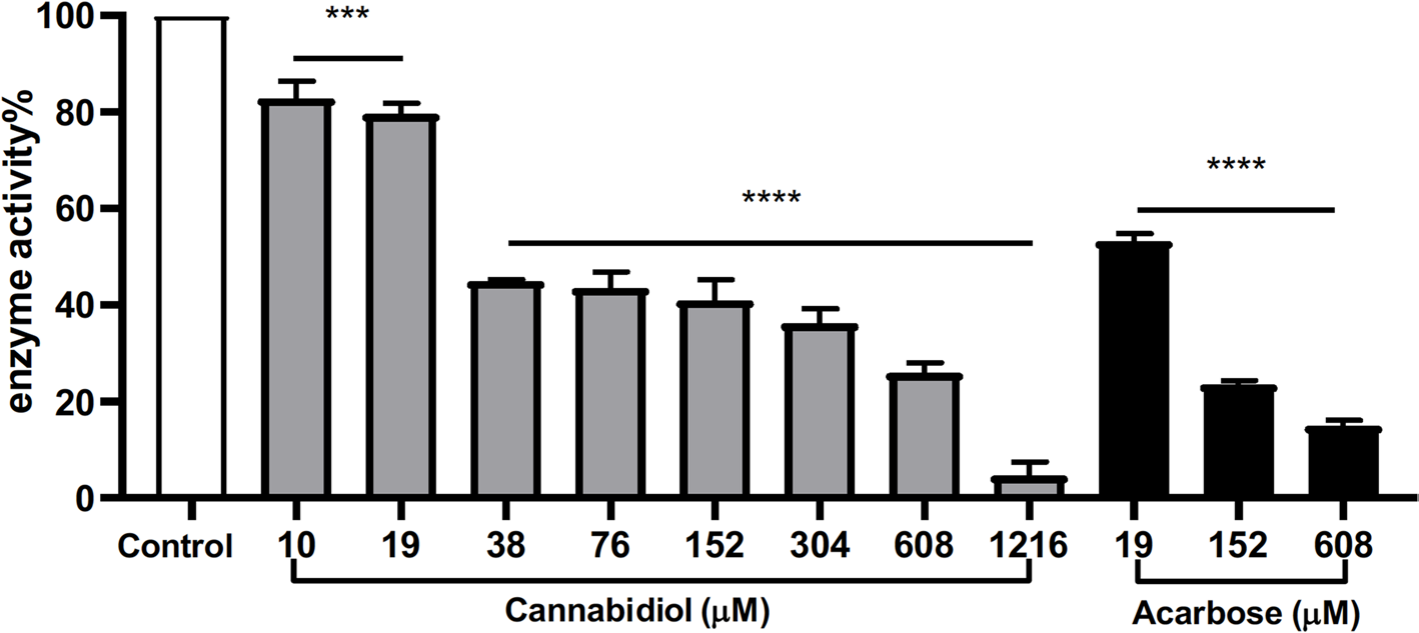
Inhibitory effect of CBD on *α*-glucosidase activity at concentrations of 10, 19, 38, 76, 152, 304, 608, and 1216 μM. Acarbose (19, 152, and 608 μM) was used as a positive control. Each sample was tested for three times, each in three replicates (*n* = 3). A *p-*value < 0.001 (***) or 0.0001 (****) was considered as statistically significant between the control and experimental groups

**Fig. 2:**
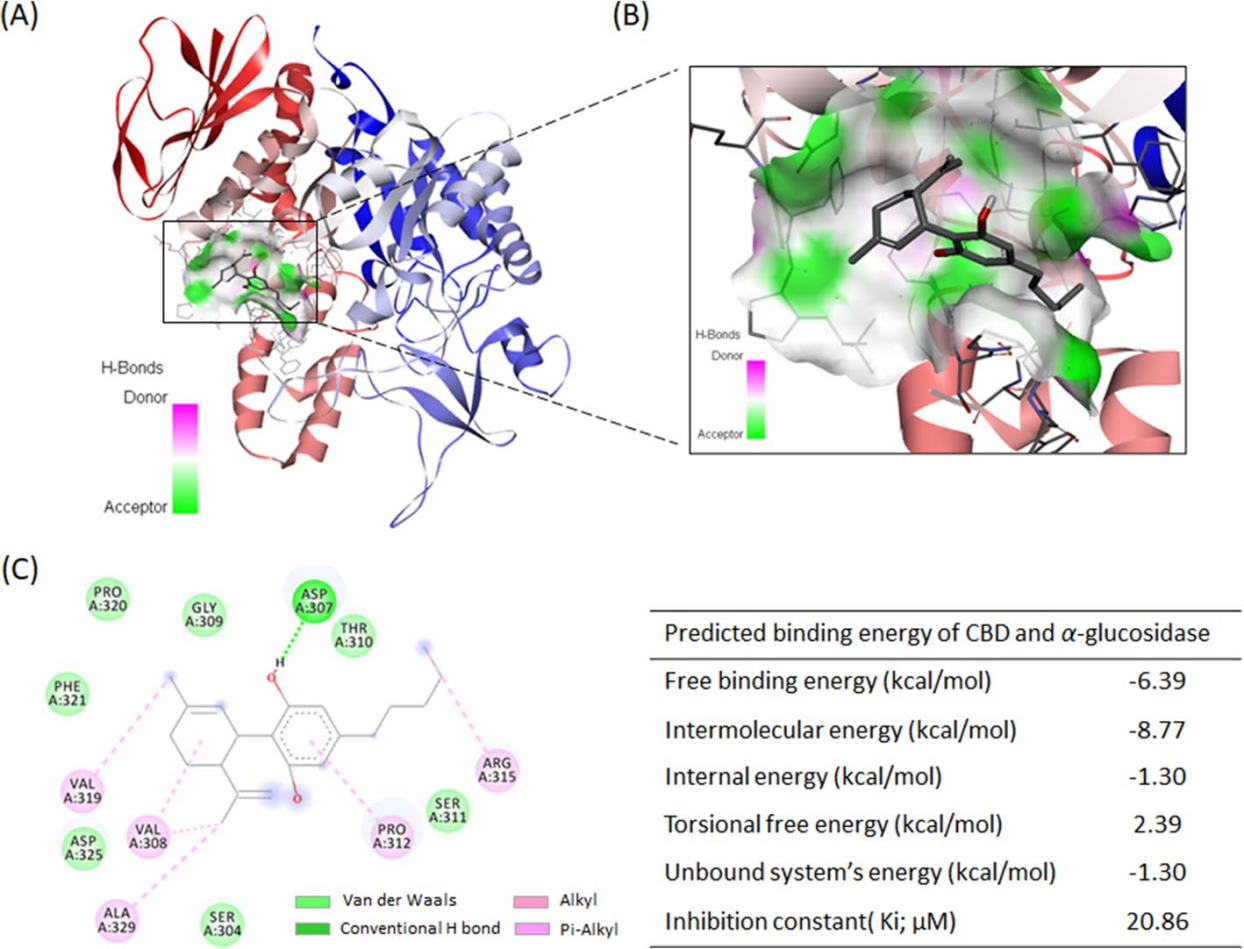
Molecular docking illustrates the interactions between CBD and *α*-glucosidase protein. Predicted binding location of CBD in *α*-glucosidase protein (**A**) and enlarged view (**B**). Molecular forces formed between CBD and *α*-glucosidase protein and predicted binding energies (**C**)

**Fig. 3:**
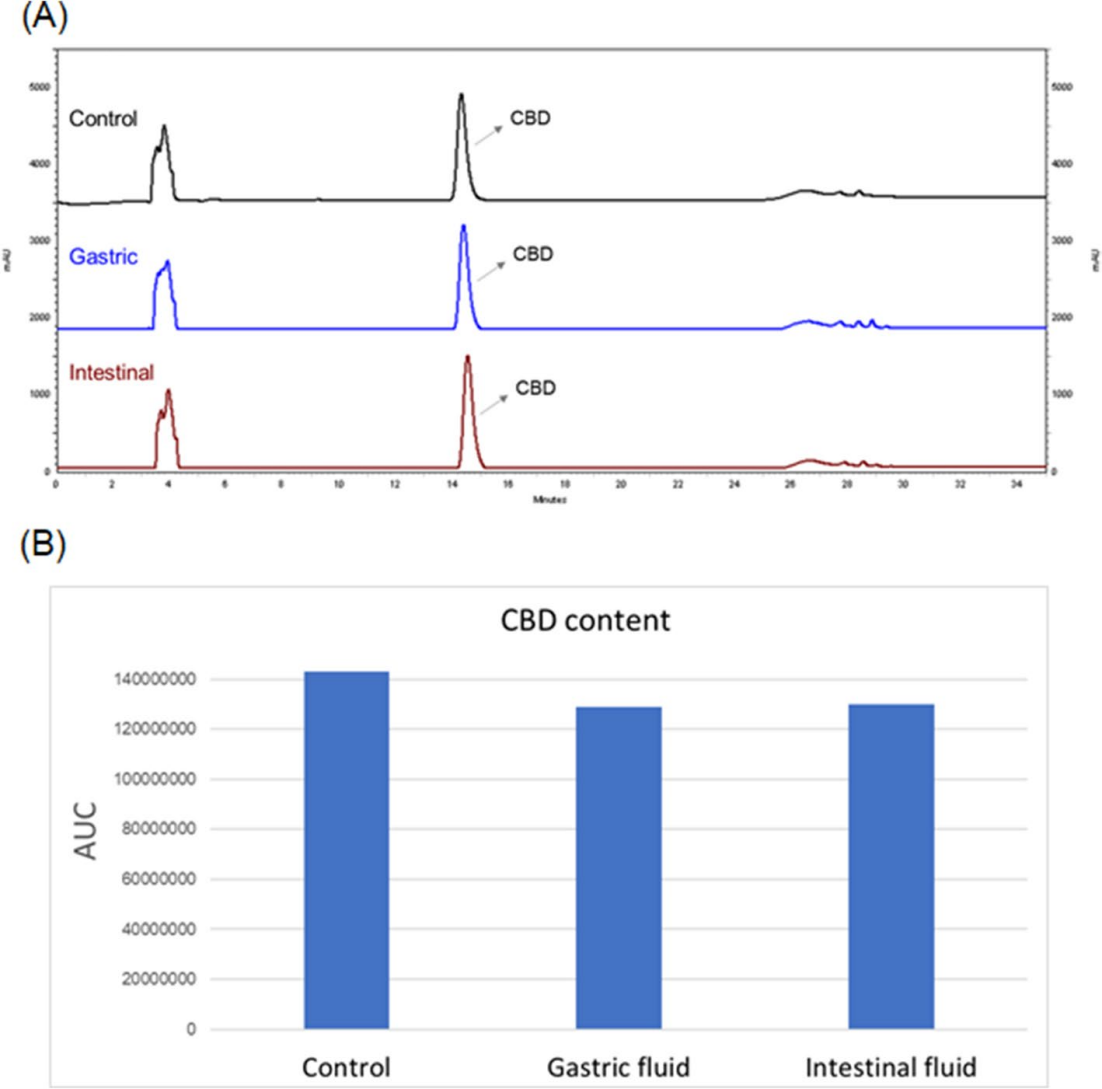
HPLC profiles of CBD incubated in phosphate buffer and in simulated gastric or intestinal fluid (**A**). CBD content (as AUC value) in control and in experimental groups (simulated gastric or intestinal fluid) for two hours (**B**). No significant statistical differences were found between CBD levels in the control and experimental groups in gastric or intestinal fluid

CBD showed moderate inhibitory effect on the activity of yeast *α*-glucosidase enzyme (Fig. 1). CBD at concentrations of 10, 19, 38, 76, 152, 304, 608, and 1216 μM had inhibition rates of 17.1, 20.4, 48.1, 56.6, 59.1, 63.7, 74.1, and 95.4%, respectively, with an IC_50_ value of 65.03 μM. At the higher concentrations (608 and 1216 μM), CBD’s anti-*α*-glucosidase activity was comparable to the positive control, acarbose (a clinical *α*-glucosidase inhibitor drug) with inhibition rates of 46.8, 76.5, and 85.1% at concentrations of 19, 152, and 608 μM, respectively. The inhibitory effect of CBD on yeast *α*-glucosidase enzyme activity was supported by molecular docking. The binding site of CBD was predicted to be at several helix and coil structures of *α*-glucosidase protein (Figs. 2 A and B). The interactions between CBD and *α*-glucosidase were primarily driven by the formation of hydrogen bond (between hydroxyl group and amino acid residue aspartic acid 307) and π-alkyl bond (between benzyl ring and proline 312) (Fig. 2 C). Several molecular forces including alkyl bond and Van der Waals were also involved in the binding between CBD and *α*-glucosidase (Fig. 2 C). The overall free binding energy of CBD and *α*-glucosidase was -6.39 kcal/mol as calculated by a sum of intermolecular energy, internal energy, torsional free energy, and unbound system’s energy (Fig. 2 C). In addition, given that *α*-glucosidase inhibitors including CBD would have to survive gastrointestinal tract digestion to exert their desired inhibitory effects on *α*-glucosidase, the stability of CBD was also evaluated in simulated gastric and intestinal fluids. HPLC analysis showed that the area under curve (AUC) vale of CBD was 142,944,597, 128,856,052, and 129,986,500 in the control group and in the experimental groups including simulated gastric and intestinal fluids, respectively. CBD was fairly stable in both simulated gastric and intestinal fluids for two hours with a remaining CBD level of 90.1 and 90.9%, respectively, as compared to the control group (Fig. 3).

A large number of *α*-glucosidase inhibitors are derived from medicinal plants and functional foods and their mechanisms of action have been reported (Kumar et al. 2011; Yin et al. 2014). Although cannabis extracts and their phytocannabinoids have been reported to show alleviative effects against diabetic conditions (Stanley et al. 2013; Weiss et al. 2008, 2006), it is not clear whether these effects are, at least partially, attributed to their inhibitory effects on *α*-glucosidase. A previously reported study showed that the phytocannabinoid, tetrahydrocannabinol, inhibited the activity of several fructose metabolism related enzymes including *α*-glucosidase, acid phosphatase, and fructose-6-phosphatase in testis, prostate and epididymis in a rat model (du Plessis et al. 2015). To the best of our knowledge, this is the first report of the inhibitory effect of CBD on *α*-glucosidase activity which was supported by molecular docking data. However, a limitation of the current study is that the *α*-glucosidase is obtained from *Saccharomyces cerevisiae* (yeast *α*-glucosidase). Although yeast *α*-glucosidase enzyme is a well-accepted experimental model to evaluate the potency of *α*-glucosidase inhibitors (Hakamata et al. 2009), studies suggest that some *α*-glucosidase inhibitors showed weaker activity when they are evaluated in assays with *α*-glucosidase obtained from mammalian sources (Yuan et al. 2013). Nevertheless, it should be noted that CBD at the higher test concentrations (i.e. 608 and 1216 μM) in the yeast *α*-glucosidase enzymatic assay showed promising anti-*α*-glucosidase activity but its efficacy as a *α*-glucosidase inhibitor in animals or human remains unknown. Therefore, further evaluations of CBD’s inhibitory effects on *α*-glucosidase using cellular and in vivo models are warranted. In addition, given that there are hundreds of phytocannabinoids in cannabis, they may also exert inhibitory effects on *α*-glucosidase enzyme and therefore investigations of ‘whole extracts’, beyond just CBD alone, is warranted. This is also important since other ‘non-phytocannabinoids’ types of compounds in cannabis extracts may also contribute to their overall anti-glycemia effects via the modulation of *α*-glucosidase enzyme activity. For instance, flavonoids including apigenin, luteolin, quercetin, and kaempferol have been identified in cannabis extracts and they have been reported as potent *α*-glucosidase inhibitors (Proença et al. 2017).

A factor that could play an important role in CBD’s anti-*α*-glucosidase activity in vivo is its stability in a physiologically relevant environment. This is because *α*-glucosidase enzymes are secreted in the brush border of the small intestine and CBD would have to survive drastic pH conditions, including acidic gastric and basic intestinal environments, to reach the small intestine for desired biological functions. Our current study supported that CBD is fairly stable after exposure to simulated gastric and intestinal fluids for two hours. This is in agreement with previously reported pharmacokinetic studies showing that CBD did not convert to tetrahydrocannabinol in humans (Crippa et al. 2020; Nahler et al. 2017). However, further CBD bioavailability and metabolism studies using in vivo models are needed to confirm this. Apart from the small intestine, *α*-glucosidase is also expressed in other organs including lung and epididymis at lower levels (Peña et al. 2004; de Vries et al. 1985). Thus, its bioavailability in different tissues may vary given that CBD may be consumed in several forms (e.g. oral intake and inhalation). Therefore, further studies are warranted to elucidate the pharmacokinetic profiles of CBD consumed or administered via different routes. This is critical for CBD consumers to achieve desirable effects and to avoid potential side effects. Another limitation of the current study is that the concentration of CBD used in the stability study (636 μM) may not reflect its bioavailability in physiological relevant conditions. In addition, the stability study was performed using simulated biological fluids, which may also not be physiologically relevant. Although the artificial fluids-based experiments in our study provided useful preliminary data on the stability of CBD, findings from the current study need to be confirmed by further stability studies using isolated stomach or intestine of animal models (e.g. mouse or rat).

In summary, CBD showed moderate inhibitory effect on the activity of yeast *α*-glucosidase enzyme, which was supported by molecular docking experiments. In addition, CBD was stable in simulated gastric and intestinal fluids for two hours. Further cellular and in vivo studies are warranted to evaluate CBD’s anti-*α*-glucosidase and anti-hyperglycemia activity. Overall, the findings from this study add to the growing body of evidence supporting the potential use of CBD for type II diabetes management.

## Data Availability

The data used for this study are available from the corresponding author with a reasonable request.

## Abbreviations

AUC: Area under curve
CBD: Cannabidiol
DMSO: Dimethyl sulfoxide
HPLC: High-performance liquid chromatography
PDB: Protein data base
*p*NPG: *p*-Nitrophenyl-α-D-glucopyranoside
